# 2-Chloro-*N*-(3,5-dichloro­phenyl)­benzamide

**DOI:** 10.1107/S1600536808018072

**Published:** 2008-06-19

**Authors:** B. Thimme Gowda, Sabine Foro, B. P. Sowmya, Hartmut Fuess

**Affiliations:** a Department of Chemistry, Mangalore University, Mangalagangotri 574 199, Mangalore, India; b Institute of Materials Science, Darmstadt University of Technology, Petersenstrasse 23, D-64287 Darmstadt, Germany

## Abstract

The amide group in the structure of the title compound (N35DCP2CBA), C_13_H_8_Cl_3_NO, is *trans*-planar, similar to that observed in *N*-(3-chloro­phen­yl)benzamide, *N*-(3,5-dichloro­phen­yl)benzamide, 2-chloro-*N*-phenyl­benzamide and other benzanilides. The C=O bond in N35DCP2CBA is *anti* to the *ortho*-chloro substituent in the benzoyl ring. The amide group makes dihedral angles of 63.1 (12) and 31.1 (17)°, respectively, with the benzoyl and aniline benzene rings, while the dihedral angle between the two benzene rings is 32.1 (2)°. The mol­ecules are linked into chains along the *b* axis by N—H⋯O hydrogen bonds.

## Related literature

For related literature, see: Gowda *et al.* (2003 ▶); Gowda, Foro *et al.* (2008 ▶); Gowda, Tokarčík *et al.* (2008 ▶).
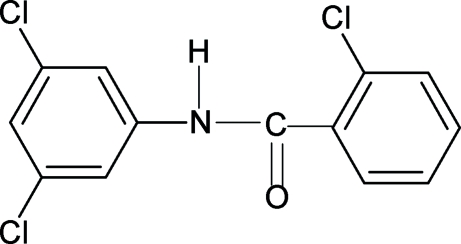


## Experimental

### 

#### Crystal data

C_13_H_8_Cl_3_NO
*M* *_r_* = 300.55
Orthorhombic, 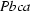

*a* = 14.699 (1) Å
*b* = 8.736 (1) Å
*c* = 20.445 (2) Å
*V* = 2625.4 (4) Å^3^
*Z* = 8
Mo *K*α radiation
μ = 0.68 mm^−1^
*T* = 299 (2) K
0.38 × 0.14 × 0.06 mm

#### Data collection

Oxford Diffraction Xcalibur diffractometer
Absorption correction: multi-scan (*CrysAlis RED*; Oxford Diffraction, 2007 ▶) *T*_min_ = 0.781, *T*_max_ = 0.960
12954 measured reflections
2686 independent reflections
1288 reflections with *I* > 2σ(*I*)
*R*_int_ = 0.094

#### Refinement

*R*[*F* ^2^ > 2σ(*F* ^2^)] = 0.056
*wR*(*F* ^2^) = 0.230
*S* = 1.08
2686 reflections
166 parameters
H atoms treated by a mixture of independent and constrained refinement
Δρ_max_ = 0.45 e Å^−3^
Δρ_min_ = −0.34 e Å^−3^

### 

Data collection: *CrysAlis CCD* (Oxford Diffraction, 2007 ▶); cell refinement: *CrysAlis RED* (Oxford Diffraction, 2007 ▶); data reduction: *CrysAlis RED*; program(s) used to solve structure: *SHELXS97* (Sheldrick, 2008 ▶); program(s) used to refine structure: *SHELXL97* (Sheldrick, 2008 ▶); molecular graphics: *PLATON* (Spek, 2003 ▶); software used to prepare material for publication: *SHELXL97*.

## Supplementary Material

Crystal structure: contains datablocks I, global. DOI: 10.1107/S1600536808018072/ci2616sup1.cif

Structure factors: contains datablocks I. DOI: 10.1107/S1600536808018072/ci2616Isup2.hkl

Additional supplementary materials:  crystallographic information; 3D view; checkCIF report

## Figures and Tables

**Table 1:** Hydrogen-bond geometry (Å, °)

*D*—H⋯*A*	*D*—H	H⋯*A*	*D*⋯*A*	*D*—H⋯*A*
N1—H1N⋯O1^i^	0.81 (5)	2.14 (5)	2.913 (5)	160 (5)

Symmetry code: (i) 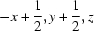.

**Fig. 1.**
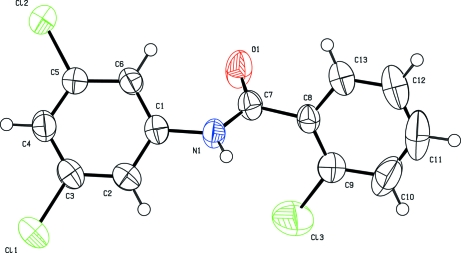
Molecular structure of the title compound, showing the atom labelling scheme. Displacement ellipsoids are drawn at the 50% probability level. H atoms are represented as small spheres of arbitrary radii.

**Fig. 2.**
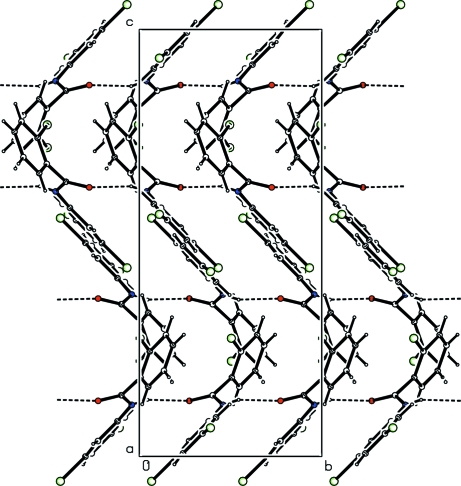
Molecular packing of the title compound, viewed along the a axis. Hydrogen bonds are shown as dashed lines.

## supplementary crystallographic information

### Comment

In the present work, the structure of 2-chloro-*N*-(3,5-dichlorophenyl)-benzamide (N35DCP2CBA) has been determined to explore the effect of substituents on the structure of benzanilides (Gowda *et al.*, 2003; Gowda, Foro *et al.*, 2008; Gowda, Tokarčík *et al.*, 2008). The N—H and C═O bonds in the amide group of N35DCP2CBA are *trans* to each other (Fig.1), similar to that observed in *N*-(3-chlorophenyl)-benzamide(N3CPBA) (Gowda, Tokarčík *et al.*, 2008), *N*-(3,5-dichlorophenyl)-benzamide (N35DCPBA) (Gowda, Foro *et al.*, 2008), 2-chloro-*N*-(phenyl)-benzamide (NP2CBA) (Gowda *et al.*, 2003) and other benzanilides. Further, the conformation of the C═O bond in the structure of N35DCP2CBA is *anti* to the *ortho*-chloro substituent in the benzoyl ring, compared to the *syn* conformation observed in NP2CBA. The amide group –NHCO– makes dihedral angles of 63.1 (12)° and 31.1 (17)° with the benzoyl and aniline rings, respectively, while the two benzene rings (benzoyl and aniline) form a dihedral angle of 32.1 (2)°, compared to the corresponding values of 14.3 (8)°, 44.4 (4)° and 58.3 (1)° in N35DCPBA.

In the crystal structure, the molecules are linked by N—H···O hydrogen bonds (Table 1) forming chains running along the *a* axis, as shown in Fig. 2.

### Experimental

The title compound was prepared according to the literature method (Gowda *et al.*, 2003). The purity of the compound was checked by determining its melting point. It was characterized by recording its infrared and NMR spectra. Single crystals of the title compound were obtained from an ethanolic solution and used for X-ray diffraction studies at room temperature.

### Refinement

The N-bound H atom was located in a difference map, and its positional parameters were refined [N—H = 0.81 (5) Å]. C-bound H atoms were positioned geometrically and refined using a riding model with C—H = 0.93 Å. All H atoms were refined with *U*_iso_(H) = 1.2*U*_eq_(parent atom).

### Crystal data

**Table:**

C_13_H_8_Cl_3_NO	*F*_000_ = 1216
*M**_r_* = 300.55	*D*_x_ = 1.521 Mg m^−^^3^
Orthorhombic, *P**b**c**a*	Mo *K*α radiation λ = 0.71073 Å
Hall symbol: -P 2ac 2ab	Cell parameters from 2008 reflections
*a* = 14.699 (1) Å	θ = 2.3–28.0º
*b* = 8.736 (1) Å	µ = 0.68 mm^−^^1^
*c* = 20.445 (2) Å	*T* = 299 (2) K
*V* = 2625.4 (4) Å^3^	Needle, colourless
*Z* = 8	0.38 × 0.14 × 0.06 mm

### Data collection

**Table:**

Oxford Diffraction Xcalibur diffractometer	2686 independent reflections
Radiation source: fine-focus sealed tube	1288 reflections with *I* > 2σ(*I*)
Monochromator: graphite	*R*_int_ = 0.094
*T* = 299(2) K	θ_max_ = 26.4º
Rotation method using ω and φ scans	θ_min_ = 2.4º
Absorption correction: multi-scan(CrysAlis RED; Oxford Diffraction, 2007)	*h* = −16→18
*T*_min_ = 0.781, *T*_max_ = 0.960	*k* = −10→10
12954 measured reflections	*l* = −25→25

### Refinement

**Table:**

Refinement on *F*^2^	Secondary atom site location: difference Fourier map
Least-squares matrix: full	Hydrogen site location: inferred from neighbouring sites
*R*[*F*^2^ > 2σ(*F*^2^)] = 0.056	H atoms treated by a mixture of independent and constrained refinement
*wR*(*F*^2^) = 0.230	*w* = 1/[σ^2^(*F*_o_^2^) + (0.12*P*)^2^] where *P* = (*F*_o_^2^ + 2*F*_c_^2^)/3
*S* = 1.09	(Δ/σ)_max_ = 0.001
2686 reflections	Δρ_max_ = 0.45 e Å^−^^3^
166 parameters	Δρ_min_ = −0.34 e Å^−^^3^
Primary atom site location: structure-invariant direct methods	Extinction correction: none

### Special details

**Table:**

Geometry. All e.s.d.'s (except the e.s.d. in the dihedral angle between two l.s. planes) are estimated using the full covariance matrix. The cell e.s.d.'s are taken into account individually in the estimation of e.s.d.'s in distances, angles and torsion angles; correlations between e.s.d.'s in cell parameters are only used when they are defined by crystal symmetry. An approximate (isotropic) treatment of cell e.s.d.'s is used for estimating e.s.d.'s involving l.s. planes.
Refinement. Refinement of *F*^2^ against ALL reflections. The weighted *R*-factor *wR* and goodness of fit *S* are based on *F*^2^, conventional *R*-factors *R* are based on *F*, with *F* set to zero for negative *F*^2^. The threshold expression of *F*^2^ > σ(*F*^2^) is used only for calculating *R*-factors(gt) *etc*. and is not relevant to the choice of reflections for refinement. *R*-factors based on *F*^2^ are statistically about twice as large as those based on *F*, and *R*- factors based on ALL data will be even larger.

### Fractional atomic coordinates and isotropic or equivalent isotropic displacement parameters (Å^2^)

**Table:**

	*x*	*y*	*z*	*U*_iso_*/*U*_eq_	
Cl1	0.60823 (8)	0.38987 (17)	0.43298 (7)	0.0724 (5)	
Cl2	0.35071 (9)	0.06746 (18)	0.55821 (7)	0.0769 (5)	
Cl3	0.27012 (13)	0.5045 (3)	0.22328 (10)	0.1267 (9)	
O1	0.1652 (2)	0.2743 (4)	0.36978 (19)	0.0721 (11)	
N1	0.2723 (2)	0.4586 (4)	0.3792 (2)	0.0479 (10)	
H1N	0.284 (3)	0.545 (6)	0.367 (2)	0.057*	
C1	0.3381 (3)	0.3802 (5)	0.4176 (2)	0.0444 (10)	
C2	0.4295 (3)	0.4206 (5)	0.4086 (2)	0.0500 (11)	
H2	0.4460	0.4947	0.3782	0.060*	
C3	0.4950 (3)	0.3468 (5)	0.4463 (2)	0.0533 (12)	
C4	0.4722 (3)	0.2395 (5)	0.4929 (2)	0.0551 (12)	
H4	0.5165	0.1927	0.5185	0.066*	
C5	0.3819 (3)	0.2042 (5)	0.5002 (2)	0.0485 (11)	
C6	0.3138 (3)	0.2729 (5)	0.4639 (2)	0.0455 (10)	
H6	0.2531	0.2474	0.4706	0.055*	
C7	0.1939 (3)	0.4018 (5)	0.3566 (2)	0.0463 (11)	
C8	0.1385 (3)	0.5072 (4)	0.3149 (2)	0.0421 (10)	
C9	0.1654 (3)	0.5573 (6)	0.2540 (3)	0.0619 (13)	
C10	0.1078 (5)	0.6459 (7)	0.2155 (3)	0.090 (2)	
H10	0.1259	0.6788	0.1742	0.108*	
C11	0.0241 (5)	0.6837 (7)	0.2398 (4)	0.094 (2)	
H11	−0.0148	0.7439	0.2148	0.113*	
C12	−0.0030 (4)	0.6354 (7)	0.2991 (4)	0.0825 (18)	
H12	−0.0604	0.6622	0.3144	0.099*	
C13	0.0520 (3)	0.5485 (5)	0.3367 (3)	0.0577 (13)	
H13	0.0320	0.5158	0.3775	0.069*	

### Atomic displacement parameters (Å^2^)

**Table:**

	*U*^11^	*U*^22^	*U*^33^	*U*^12^	*U*^13^	*U*^23^
Cl1	0.0387 (7)	0.0917 (10)	0.0867 (11)	−0.0057 (6)	−0.0020 (6)	0.0066 (8)
Cl2	0.0570 (8)	0.0977 (11)	0.0759 (10)	0.0016 (7)	−0.0032 (7)	0.0413 (8)
Cl3	0.0856 (14)	0.213 (3)	0.0812 (13)	0.0195 (13)	0.0327 (10)	0.0204 (14)
O1	0.070 (2)	0.0475 (19)	0.098 (3)	−0.0128 (16)	−0.037 (2)	0.0190 (19)
N1	0.044 (2)	0.0401 (19)	0.060 (3)	−0.0031 (17)	−0.0158 (18)	0.0114 (18)
C1	0.042 (2)	0.044 (2)	0.047 (3)	0.0060 (19)	−0.0056 (19)	−0.004 (2)
C2	0.042 (3)	0.053 (3)	0.055 (3)	−0.004 (2)	0.001 (2)	−0.004 (2)
C3	0.039 (3)	0.060 (3)	0.061 (3)	−0.004 (2)	−0.008 (2)	−0.005 (3)
C4	0.046 (3)	0.068 (3)	0.052 (3)	0.010 (2)	−0.009 (2)	0.003 (3)
C5	0.044 (2)	0.052 (2)	0.050 (3)	0.002 (2)	0.000 (2)	0.008 (2)
C6	0.035 (2)	0.047 (2)	0.055 (3)	0.0028 (19)	−0.002 (2)	0.004 (2)
C7	0.043 (3)	0.041 (2)	0.055 (3)	0.0052 (19)	−0.007 (2)	−0.002 (2)
C8	0.039 (2)	0.041 (2)	0.046 (3)	−0.0013 (17)	−0.0121 (19)	−0.002 (2)
C9	0.054 (3)	0.074 (3)	0.057 (3)	−0.001 (2)	−0.005 (2)	0.009 (3)
C10	0.111 (6)	0.095 (5)	0.065 (4)	−0.004 (4)	−0.030 (4)	0.028 (4)
C11	0.099 (6)	0.061 (4)	0.121 (7)	0.012 (3)	−0.065 (5)	0.005 (4)
C12	0.062 (4)	0.083 (4)	0.103 (5)	0.026 (3)	−0.027 (4)	−0.017 (4)
C13	0.048 (3)	0.059 (3)	0.066 (3)	0.010 (2)	−0.011 (2)	−0.007 (3)

### Geometric parameters (Å, °)

**Table:**

Cl1—C3	1.728 (5)	C5—C6	1.383 (6)
Cl2—C5	1.744 (5)	C6—H6	0.93
Cl3—C9	1.725 (6)	C7—C8	1.496 (6)
O1—C7	1.221 (5)	C8—C9	1.376 (7)
N1—C7	1.338 (6)	C8—C13	1.396 (6)
N1—C1	1.422 (5)	C9—C10	1.392 (8)
N1—H1N	0.81 (5)	C10—C11	1.368 (9)
C1—C6	1.380 (6)	C10—H10	0.93
C1—C2	1.400 (6)	C11—C12	1.344 (9)
C2—C3	1.392 (6)	C11—H11	0.93
C2—H2	0.93	C12—C13	1.350 (7)
C3—C4	1.378 (6)	C12—H12	0.93
C4—C5	1.371 (6)	C13—H13	0.93
C4—H4	0.93		
			
C7—N1—C1	126.7 (4)	O1—C7—N1	124.1 (4)
C7—N1—H1N	115 (3)	O1—C7—C8	119.9 (4)
C1—N1—H1N	118 (4)	N1—C7—C8	115.9 (4)
C6—C1—C2	120.7 (4)	C9—C8—C13	117.9 (4)
C6—C1—N1	122.0 (4)	C9—C8—C7	123.7 (4)
C2—C1—N1	117.3 (4)	C13—C8—C7	118.2 (4)
C3—C2—C1	118.3 (4)	C8—C9—C10	120.9 (5)
C3—C2—H2	120.9	C8—C9—Cl3	120.0 (4)
C1—C2—H2	120.9	C10—C9—Cl3	119.0 (5)
C4—C3—C2	122.0 (4)	C11—C10—C9	118.5 (6)
C4—C3—Cl1	119.4 (4)	C11—C10—H10	120.8
C2—C3—Cl1	118.5 (4)	C9—C10—H10	120.8
C5—C4—C3	117.6 (4)	C12—C11—C10	121.2 (5)
C5—C4—H4	121.2	C12—C11—H11	119.4
C3—C4—H4	121.2	C10—C11—H11	119.4
C4—C5—C6	123.0 (4)	C11—C12—C13	120.8 (6)
C4—C5—Cl2	118.8 (3)	C11—C12—H12	119.6
C6—C5—Cl2	118.1 (3)	C13—C12—H12	119.6
C1—C6—C5	118.4 (4)	C12—C13—C8	120.6 (5)
C1—C6—H6	120.8	C12—C13—H13	119.7
C5—C6—H6	120.8	C8—C13—H13	119.7
			
C7—N1—C1—C6	−35.1 (7)	O1—C7—C8—C9	−115.6 (6)
C7—N1—C1—C2	147.4 (5)	N1—C7—C8—C9	66.7 (6)
C6—C1—C2—C3	1.6 (6)	O1—C7—C8—C13	59.7 (6)
N1—C1—C2—C3	179.1 (4)	N1—C7—C8—C13	−118.0 (5)
C1—C2—C3—C4	−1.8 (7)	C13—C8—C9—C10	−0.2 (7)
C1—C2—C3—Cl1	177.2 (3)	C7—C8—C9—C10	175.1 (5)
C2—C3—C4—C5	1.5 (7)	C13—C8—C9—Cl3	−177.8 (4)
Cl1—C3—C4—C5	−177.5 (4)	C7—C8—C9—Cl3	−2.5 (6)
C3—C4—C5—C6	−1.1 (7)	C8—C9—C10—C11	0.7 (9)
C3—C4—C5—Cl2	179.4 (3)	Cl3—C9—C10—C11	178.3 (5)
C2—C1—C6—C5	−1.2 (6)	C9—C10—C11—C12	−0.7 (10)
N1—C1—C6—C5	−178.6 (4)	C10—C11—C12—C13	0.3 (9)
C4—C5—C6—C1	1.0 (7)	C11—C12—C13—C8	0.2 (8)
Cl2—C5—C6—C1	−179.5 (3)	C9—C8—C13—C12	−0.2 (7)
C1—N1—C7—O1	4.9 (8)	C7—C8—C13—C12	−175.8 (4)
C1—N1—C7—C8	−177.5 (4)		

### Hydrogen-bond geometry (Å, °)

**Table:**

*D*—H···*A*	*D*—H	H···*A*	*D*···*A*	*D*—H···*A*
N1—H1N···O1^i^	0.81 (5)	2.14 (5)	2.913 (5)	160 (5)

Symmetry codes: (i) −*x*+1/2, *y*+1/2, *z*.
